# Chronic viral infection aggravates white adipose tissue dysfunction and liver pathology in obesity

**DOI:** 10.1016/j.molmet.2026.102394

**Published:** 2026-06-09

**Authors:** Katarzyna M. Luda, Marianne Agerholm, Si Brask Sonne, Dipsikha Biswas, Lisa DeCamp, Russell G. Jones, Allan Randrup Thomsen, Benjamin Anderschou Holbeck Jensen, Jan Pravsgaard Christensen, Kei Sakamoto

**Affiliations:** 1 Novo Nordisk Foundation Center for Basic Metabolic Research, University of Copenhagen, 2200 Copenhagen, Denmark; 2 Department of Biomedical Sciences, 2200 Copenhagen, Denmark; 3 Department of Metabolism and Nutritional Programming, Van Andel Institute, Grand Rapids, MI, USA; 4 Department of Immunology and Microbiology, University of Copenhagen, 2200 Copenhagen, Denmark

**Keywords:** Obesity, White adipose tissue (WAT), viral infection, MASLD, Adipose–liver crosstalk

## Abstract

**Background:**

White adipose tissue (WAT) plays a central role in maintaining systemic metabolic homeostasis by buffering lipid flux throughout the body. Impairment of this lipid-buffering capacity is a hallmark of obesity and has also been observed during chronic viral infection. Such dysfunction is closely associated with ectopic fat accumulation, particularly in the liver.

**Objectives:**

We hypothesized that the coexistence of obesity and chronic viral infection exacerbates WAT dysfunction, thereby promoting liver pathology. However, the specific response of obese WAT to chronic viral infection – and its downstream impact on liver health – remains to be explored.

**Methods:**

To investigate this interaction, we employed a model of chronic viral infection in mice using lymphocytic choriomeningitis virus (LCMV) clone 13.

**Results:**

In obese hosts, chronic infection caused sustained WAT depletion and progressive weight loss, accompanied by a reduction of Tim-4^+^ eWAT-resident macrophages and features reminiscent of lipodystrophy and aggravated metabolic dysfunction-associated steatotic liver disease (MASLD). Depletion of CD8^+^ T cells, the key mediators of LCMV-driven weight loss in lean mice, only modestly attenuated weight loss and did not ameliorate liver pathology in obese mice. Likewise, therapeutic interventions including TNF-α blockade and glycemic control with metformin did not reverse infection-induced weight loss; moreover, TNF-α blockade failed to improve liver pathology.

**Conclusions:**

Collectively, these findings reveal a previously unrecognized crosstalk between WAT and the liver in infection-driven MASLD, highlight distinct responses in WAT of obese mice compared to their lean counterpart, and underscore the increased susceptibility to virus-induced metabolic complications in obesity.

## Highlights

• Chronic viral infection drives sustained fat loss in obese mice.
• Obese mice develop severe MASLD during infection-induced WAT depletion.
• CD8^+^ T-cell depletion in infected obese mice partly rescues weight loss, not liver pathology.

## 1 Introduction

Viral infections are recognized as contributing factors in the pathogenesis of metabolic diseases [1]. Viruses causing chronic infection are particularly important due to their capacity to induce sustained inflammation. Especially individuals with obesity - who exhibit preexisting metabolic abnormalities, low-grade inflammation, and altered antiviral responses [[2], [3], [4], [5], [6]] - are at increased risk of viral infection-induced metabolic complications. Despite these associations, the specific metabolic consequences of chronic viral infections in obesity remain insufficiently characterized.

White adipose tissue (WAT) plays a pivotal role in maintaining metabolic homeostasis and participates in the host response to infection [[7], [8], [9]]. WAT dysfunction is characterized by inflammation and reduced lipid storage, and has been linked to hepatic fat deposition contributing to the development and progression of metabolic dysfunction-associated steatotic liver disease (MASLD) [[10], [11], [12], [13], [14]]. This metabolic disease is characterized by hepatic lipid accumulation, progressing from simple steatosis to liver inflammation, and can ultimately lead to hepatic dysfunction, particularly in the presence of inflammation [15]. The liver serves as a site of replication for numerous viruses causing chronic infection, including hepatitis viruses, cytomegalovirus, Epstein–Barr virus in humans, as well as LCMV in mice [[16], [17], [18]]. Clinically, MASLD is a well-established comorbidity present in patients with chronic viral infections [19,20]. Notably, weight-reduction interventions have been shown to ameliorate steatosis and liver damage in these settings, highlighting the pivotal role of adipose-liver crosstalk in the progression of virus-driven liver disease [19]. These findings underscore a strong association between chronic viral infection and liver disease severity, with obesity acting as a key risk factor that may potentiate infection-associated liver pathology. However, the interplay between chronic viral infection, obese WAT, and liver disease remains incompletely understood and warrant further investigation.

To investigate this interplay, we used the chronic LCMV clone 13 model, known to induce liver pathology in mice [21,22]. We examined the interaction between chronic infection and obesity, with a particular focus on inguinal (i) WAT and epididymal (e) WAT function and the progression of MASLD. We assessed whether previously described immunological regulators of WAT function, such as TNF-α, CD8^+^ T cells as well as impaired glucose regulation [7,9,23,24], affected WAT homeostasis in infected obese mice. Finally, we analyzed macrophages, which are recognized as key regulators of WAT homeostasis [25,26]. We hypothesized that chronic viral infection intensifies WAT impairment in obesity and may be associated with increased lipid mobilization from WAT to the liver, along with heightened inflammation, potentially contributing to the progression of pre-existing MASLD. This study elucidates how chronic infection may influence lipid metabolism across tissues, potentially contributing to MASLD progression in obesity.

## 2 Results and discussion

### 2.1 Chronic viral infection induces progressive weight loss associated with WAT depletion in obese mice

Given the effect of chronic viral infection on adipose tissue homoeostasis and liver pathology in lean settings [7,21], we first aimed to investigate how chronic viral infection perturbs WAT homeostasis in obesity. We studied the response of lean and high-fat diet (HFD) induced obese mice infected with the chronic virus LCMV clone 13. Male C57BL/6J mice (6 weeks old) mice were divided into two groups and fed either standard chow or HFD (60% kCal from fat) for 12–16 weeks. Prior to viral infection (at 18–22 weeks of age) mean body weight was on average 45 g in the obese group and 31 g in the lean group (Supplementary Fig. 1A).

**Figure 1:**
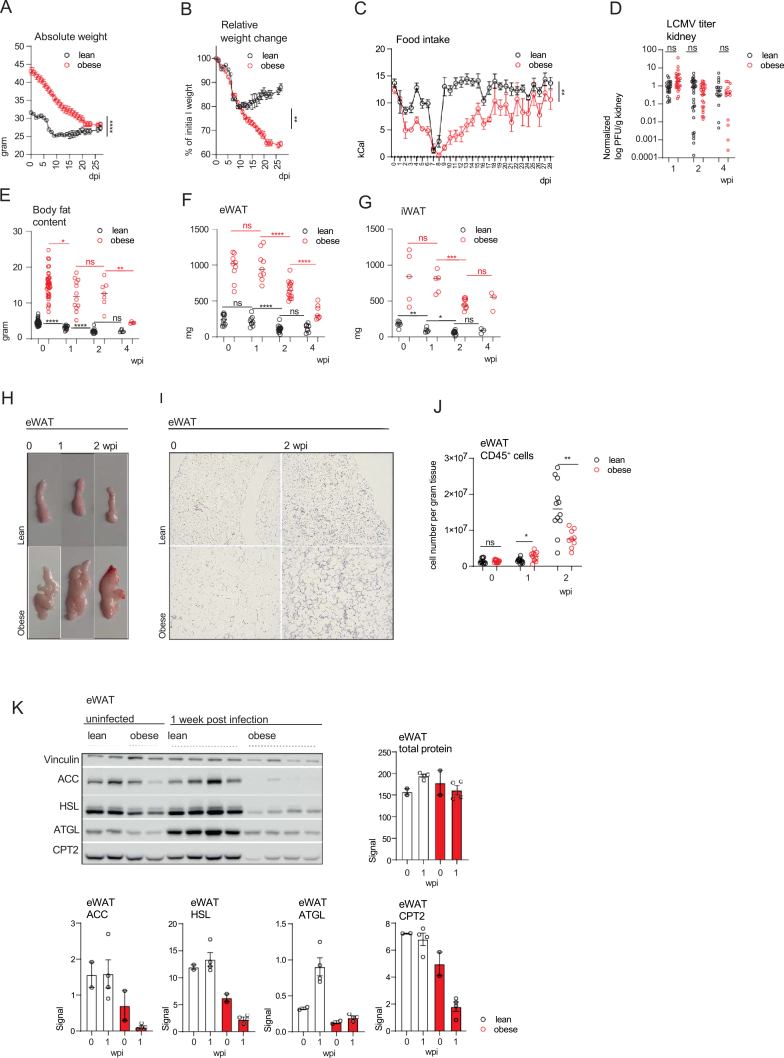
Changes in WAT induced by chronic viral infection in lean and obese mice. Comparison of indicated parameters between lean and obese mice in the course of infection. (A–B) Representative body weight trajectories over days post-infection (dpi) expressed as (A) absolute weights and (B) change in weight relative to the weight at the day of infection (day 0). (C) Daily food intake expressed in kCal/day. (D) Normalized values of viral titers in kidney, with each dot representing viral titer value divided by the mean value for LCMV titer of the lean group in the respective time point post infection. (E) Total body fat content and (F–G) total weights of (F) eWAT and (G) iWAT (one pad per mouse). (H–I) Representative (H) pictures and (I) histological evaluation assessed by H&E staining of the eWAT at 10-times magnification. (J) Flow cytometric quantification of immune cell (CD45^+^) infiltration into the eWAT. (K) Western blot (WB) analysis of eWAT lysates and quantification assessing total protein per WB lane and expression of proteins involved in lipid metabolism. dpi – days post infection, wpi – weeks post infection. Each dot represents (A–C) average value of the pool of at least five mice or (D-G, J-K) each dot and WB band represents one biological replicate. Error bars represent mean ± SEM. Statistical significance was determined using two-tailed Student's t-tests for comparisons between two groups and mixed-effects models for repeated measures analyses. ∗p < 0.05, ∗∗p < 0.01, ∗∗∗p < 0.001, ∗∗∗∗p < 0.0001, ns, not significant. Related to Supplementary Fig. 1.

Consistent with previous reports [7], LCMV clone 13 infection in lean mice induced an acute reduction in body weight that stabilized within ten days, followed by gradual recovery (Figure 1A,B). In contrast, obese mice exhibited progressive weight loss, with body weights eventually converging towards average body weight of their lean counterparts within four weeks post infection (Figure 1A,B). This indicates compromised mechanisms protecting obese mice from uncontrolled weight loss during infection. For comparison, obese ferrets infected with the acute H1N1 virus exhibit only transient weight loss [4], suggesting that the progressive weight loss observed here is a unique feature of chronic viral infection - likely driven by persistent immune activation. To determine whether the marked weight loss reflected an anorectic behavior, a common host response to infection [7,9], we monitored daily caloric intake. Obese mice had reduced food consumption during the first week of infection; however, weight loss persisted despite partial recovery of HFD intake (Figure 1C), indicating additional mechanisms beyond anorexia. Similarly, chow-fed *ob/ob* mice has been reported to exhibit sustained weight loss during persistent LCMV infection [27], suggesting that the response is driven by the obese state rather than by diet composition. Monitoring of water intake, body temperature, and activity revealed no correlation that could explain the sustained weight loss, suggesting these factors were not primary contributors (Supplementary Fig. 1B–D). While increased viral loads have been linked to enhanced tissue pathology in influenza-infected obese ferrets [4], viral loads in our study were comparable between lean and obese mice (Figure 1D), indicating that the progressive weight loss in the obese mice does not reflect an increased viral burden.

Using DEXA scanning, we quantified body fat content throughout the course of infection. Lean mice lost less total fat and showed stabilization of fat depletion after the second week (Figure 1E). The dynamics of the two major fat depots - epididymal (eWAT) and inguinal (iWAT) adipose tissues - followed a similar pattern (Figure 1F,G). In contrast, obese mice exhibited progressive fat loss ultimately reaching levels comparable to those in lean mice by week four post-infection (Figure 1E). Notably, total fat in obese mice decreased during the first week post-infection, despite unchanged eWAT and iWAT mass, indicating that initial fat loss originated primarily from other depots (Figure 1E–G). Subsequently, between weeks one and two post-infection, eWAT and iWAT mass declined, while total fat mass remained stable (Figure 1E–G), consistent with the possibility of ectopic fat redistribution. Notably, in obese mice, loss of the eWAT continued until four weeks post infection, whereas loss of the iWAT ceased earlier (Figure 1F,G). This depot-specific response likely reflects intrinsic biological differences between adipose regions [28].

Together, these findings indicate that, although overall body fat in obese mice remained stable between weeks one and two post-infection, significant reductions in eWAT and iWAT mass points towards ectopic fat deposition. This prompted us to focus on the first two weeks post-infection to better understand WAT homeostasis and liver pathology.

### 2.2 WAT displays hallmarks of lipodystrophy upon LCMV infection in obese mice

Macroscopic examination of eWAT, but not iWAT, revealed pronounced redness in the distal region, indicative of ongoing local inflammation, particularly in obese mice two weeks post-infection, while infected lean mice showed no such manifestation (Figure 1H, Supplementary Fig. 1E). Histological analysis of H&E-stained sections of the distal part of the eWAT at two weeks post infection confirmed cellular infiltrates surrounding adipocytes in both groups, with obese adipose tissue containing densely clustered infiltrating cells (Figure 1I). Flow cytometry further confirmed enhanced immune cell infiltration, where both groups exhibited increased total numbers and density of CD45^+^ cells in both eWAT and iWAT specifically at two weeks post-infection (Figure 1J, Supplementary Fig. 1F). Despite the distinct macroscopic phenotype of obese eWAT and its higher total number of CD45^+^ cells, the cell density per g of tissue at two weeks post-infection was lower than in lean mice. This may result from increased accumulation of infiltrating cells in the distal eWAT, alongside reduced accumulation, survival, or expansion of CD45^+^ cells in the other regions of the obese eWAT. In contrast to eWAT, the density of CD45^+^ cells harvested from the iWAT remained similar between lean and obese animals, indicating depot-specific response to the infection (Supplementary Fig. 1F). An immune cell type potentially involved in infection-induced eWAT pathology in obese settings are macrophages. This functionally heterogeneous population maintains adipose tissue homeostasis and adapts their function to inflammatory conditions [25,26]. Although macrophage density in eWAT was unchanged between lean and obese mice, we observed a marked reduction of tissue-resident Tim-4^+^ macrophages early on during infection in obese eWAT (Supplementary Fig. 4A–C). As Tim-4 is a receptor involved in apoptotic cell clearance and is expressed by vasculature-associated WAT macrophages [26,29,30], this reduction may impair efferocytosis of adipocytes, disrupt eWAT integrity leading to secondary necrosis and exacerbated eWAT inflammation.

Collectively, these findings suggest that obese eWAT may be particularly prone to pathological remodeling during infection, and that immune cell accumulation in WAT may contribute to fat depletion and heightened local and systemic inflammation.

The profound depletion of WAT in obese mice following viral infection could suggest alterations in lipid metabolism pathways. To investigate this, we analyzed the expression of key enzymes - HSL and ATGL, ACC as well as CPT2, that regulate lipolysis, adipogenesis and long-chain fatty acid utilization, respectively (Figure 1K, Supplementary Fig. 1G–H). To minimize the contribution of hematopoietic cells to WAT protein content, we analyzed samples at week one - a timepoint prior to significant immune cell infiltration and at the onset of WAT depletion (Figure 1F–G, J, Supplementary Fig. 1G–H). While lean mice exhibited consistent induction of ATGL in eWAT and iWAT, and HSL in iWAT, obese mice showed markedly reduced baseline levels of these enzymes and impaired upregulation following infection. Additionally, ACC and CPT2 expression levels were already reduced in obese mice prior to infection and profoundly downregulated in response to infection in eWAT and iWAT. These findings indicate that obese WAT exhibits impaired adipogenic capacity, reduced lipolytic activity, and diminished lipid utilization at baseline, with infection further exacerbating these functional deficits. However, we cannot exclude the possibility that the expression of these enzymes may differ at an earlier or later time during infection. To assess whether WAT loss in obese mice was associated with changes in brown adipose tissue (BAT) properties, we analyzed animals at week 2 post-infection, when food intake had resumed but fat mass depletion and weight loss persisted. UCP1 and CPT2 are key mediators of thermogenesis and mitochondrial fatty acid oxidation, respectively, and contribute to adiposity regulation [31,32]. Although UCP1 and CPT2 tended to increase with HFD feeding, possibly as a compensatory response, its levels were unchanged in infected obese mice, arguing against increased thermogenic activity as a driver of WAT depletion at this point (Supplementary Fig. 1I–J). However, these conclusions are limited by the relatively small sample size.

Collectively, obese WAT exhibits a distinct response to viral infection compared to lean WAT, characterized by features of lipodystrophy, including increased inflammation and reduced lipid-regulating enzyme expression - particularly profound in eWAT. The absence of appropriate upregulation of lipolytic enzymes suggests that increased lipolysis is not the main driver of obese WAT depletion. Instead, the observed changes point towards lipodystrophy as a potential underlying mechanism potentially driven by immune cells. Moreover, in inflammatory settings, lipoprotein lipase activity is known to be suppressed in adipose tissue [33], which may further contribute to WAT depletion in our model by limiting lipid uptake from circulating lipoproteins into storage depots. Collectively, these findings suggest that persistent viral infection induces a progressive decline in WAT function, aggravating its pathological state in obesity.

### 2.3 Viral infection exacerbates liver pathology in obese mice

WAT dysfunction and inflammation are closely linked to the development of MASLD, with lipid flux from WAT strongly correlating with the extent of hepatic steatosis [10,11,13]. Patients with lipodystrophy frequently develop MASLD [34], supporting the notion that virus-induced pathological remodeling of obese WAT may have deleterious downstream effects on hepatic function. Given the significant inflammation and depletion of WAT upon infection, we assessed whether this was associated with increased liver pathology.

**Figure 2:**
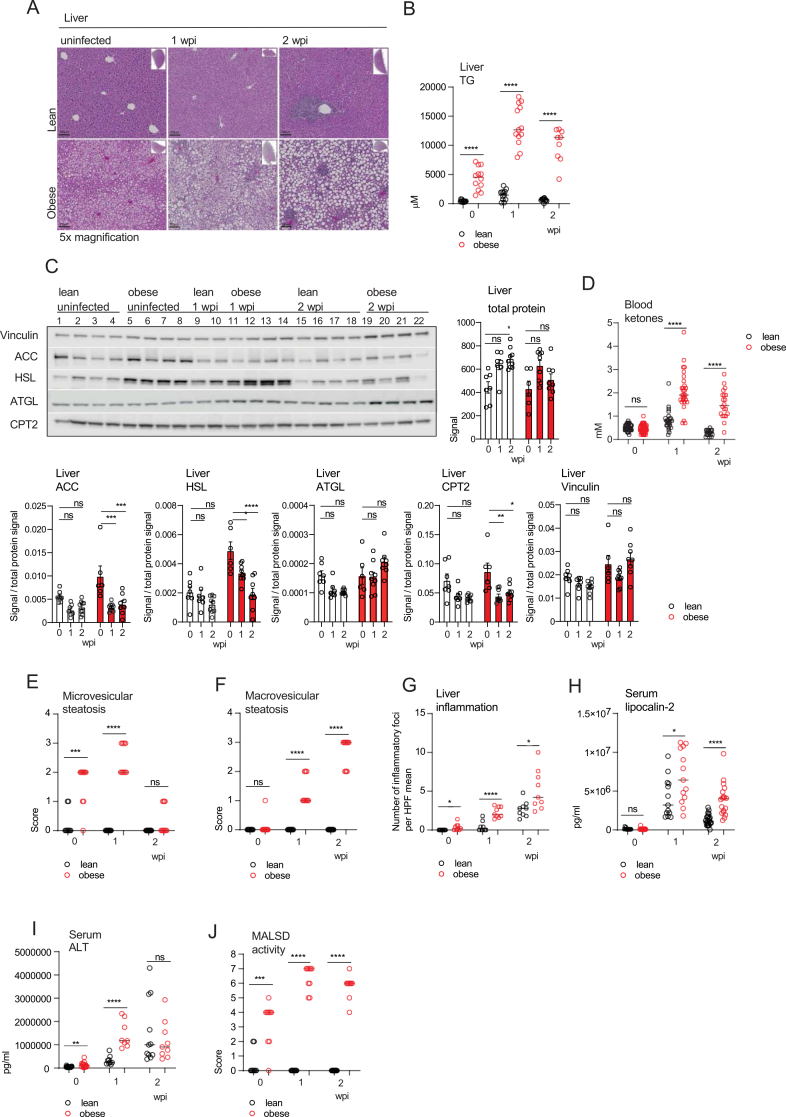
Chronic viral infection exacerbates liver pathology in obese mice. Comparison of indicated parameters between lean and obese mice in the course of infection. (A) Representative histological evaluation of liver pathology assessed by H&E-staining at 5-times magnification. Concentrations of (B) liver triglycerides (TG). (C) Representative immunoblots and quantification of total protein per WB lane, ATGL, HSL, ACC and CPT2 expression in liver tissue; signals were normalized to total protein. (D) Concentration of circulating ketone bodies. (E–G) Scoring of the H&E-stained tissues for liver pathology assessing (E) microvesicular, (F) macrovesicular steatosis and (G) hepatic immune infiltration quantified as number of inflammatory foci per high power field (HPF); (H) concentrations of lipocalin-2 and (I) alanine-aminotransferase (ALT) in serum, representing an inflammation and liver damage marker, respectively. (J) Overall MASLD activity assessed by scoring H&E-stained liver slices; wpi – weeks post infection. (B–J) Each data point and each WB band represent one biological replicate. Error bars represent mean ± SEM. Statistical significance was determined using two-tailed Student's t-tests for comparisons between two groups, for the immunoblots, statistical significance was determined by two-way ANOVA with Tukey's post hoc correction. ∗p < 0.05, ∗∗p < 0.01, ∗∗∗p < 0.001, ∗∗∗∗p < 0.0001, ns, not significant. Related to Supplementary Fig. 2.

Histological analysis of the H&E-stained liver sections revealed a progressive increase in hepatic steatosis during the first two weeks of infection in obese mice, whereas no apparent changes were observed in lean mice (Figure 2A). Nevertheless, both lean and obese mice exhibited elevated hepatic triglycerides (TG) levels post infection (Figure 2B). Although the relative increases were similar, the peak hepatic TG levels in lean mice never reached the baseline levels observed in uninfected obese mice, indicating a milder liver impact from viral infection on the liver in lean mice.

To investigate whether infection altered hepatic lipid metabolism, potentially contributing to excessive hepatic lipid accumulation in obese mice, we examined the expression of key metabolic enzymes involved in lipid metabolism in the liver (Figure 2C, Supplementary Fig. 2A). We observed modest, but not pronounced, effects of infection on the protein levels, including a trend of increased ATGL at week 2, and a reduction in HSL expression at week 1 and 2 post infection in obese mice. These changes do not indicate a coordinated upregulation of lipolysis, suggesting that altered hepatic lipolysis is unlikely to be a major driver of the infection-induced lipid accumulation observed in obese livers. We observed a reduction of ACC and CPT2 in obese livers post infection, the pattern observed in obese WAT tissue. The decrease in ACC may reflect increased influx of adipose tissue-derived fatty acids and a reduced requirement for *de novo* lipogenesis. Reduced expression of CPT2 may suggest a decreased capacity for mitochondrial fatty acid oxidation. However, more detailed functional analyses are required to draw definitive conclusions.

To assess whether impaired ketogenic capacity contributes to infection-associated hepatic steatosis, we quantified circulating ketone body levels in infected mice. Infection induced a marked surge of circulating ketone bodies, particularly in obese mice, where ketone levels remained elevated for up to two weeks post-infection (Figure 2D). In contrast, in lean mice the viral infection induced only a minor increase at one-week post-infection, which normalized in the second week. While early increases in ketones may reflect the anorectic phase of infection, the persistence of elevated ketones in obese mice despite the resumption of food intake (Figure 1C), suggests that ketogenesis acts as a compensatory adaptation to lipid excess helping to mitigate hepatic lipid accumulation.

To further evaluate the impact of infection on liver pathology, we assessed the livers histologically. Lean animals showed no steatosis throughout the infection period, whereas obese animals exhibited a transition from microvesicular dominated to macrovesicular dominated lipid accumulation (Figure 2E,F). Quantification of inflammatory foci revealed that while obese mice had negligible inflammation at baseline, they developed significant hepatic immune infiltration as early as one-week post infection (Figure 2G). In contrast, lean mice exhibited a delayed inflammatory response but reached levels comparable to those of obese mice at two weeks post-infection (Figure 2G). To assess systemic inflammation, we measured lipocaline-2 in serum, a well-established acute phase protein increased during inflammation [35]. A pronounced surge in lipocalin-2 was observed at one-week post-infection, followed by a decline at two weeks in both groups of mice (Figure 2H). However, obese mice consistently exhibited higher lipocalin-2 levels compared to their lean counterparts, suggesting a more sustained inflammatory state. In line with previous reports of LCMV-associated hepatitis LCMV induced significant ALT elevation in serum (Figure 2I) [8,36], indicative of ongoing liver damage. The ALT levels paralleled hepatic immune infiltration, peaking in obese mice at week 1 and increasing in lean mice at week 2, indicating accelerated immune-dependent liver damage in obese mice following LCMV infection (Figure 2I). Finally, although obese animals already exhibited a high MASLD activity score at baseline (Figure 2J), LCMV infection further aggravated this phenotype exclusively in obese mice supporting virus-induced ectopic lipid deposition. Finally, it has been reported that MASLD pathology correlates with loss of WAT integrity, associated with changes in WAT macrophage function [29]. This suggests that liver pathology in obese mice may be linked to infection-induced alterations in WAT macrophages (Supplementary Fig. 4).

Collectively, these results demonstrate that chronic virus-induced inflammation is associated with marked functional changes in key metabolic organs and leads to more severe liver pathology in obesity.

### 2.4 Infection-induced weight loss in obese animals is independent of TNF-α

**Figure 3:**
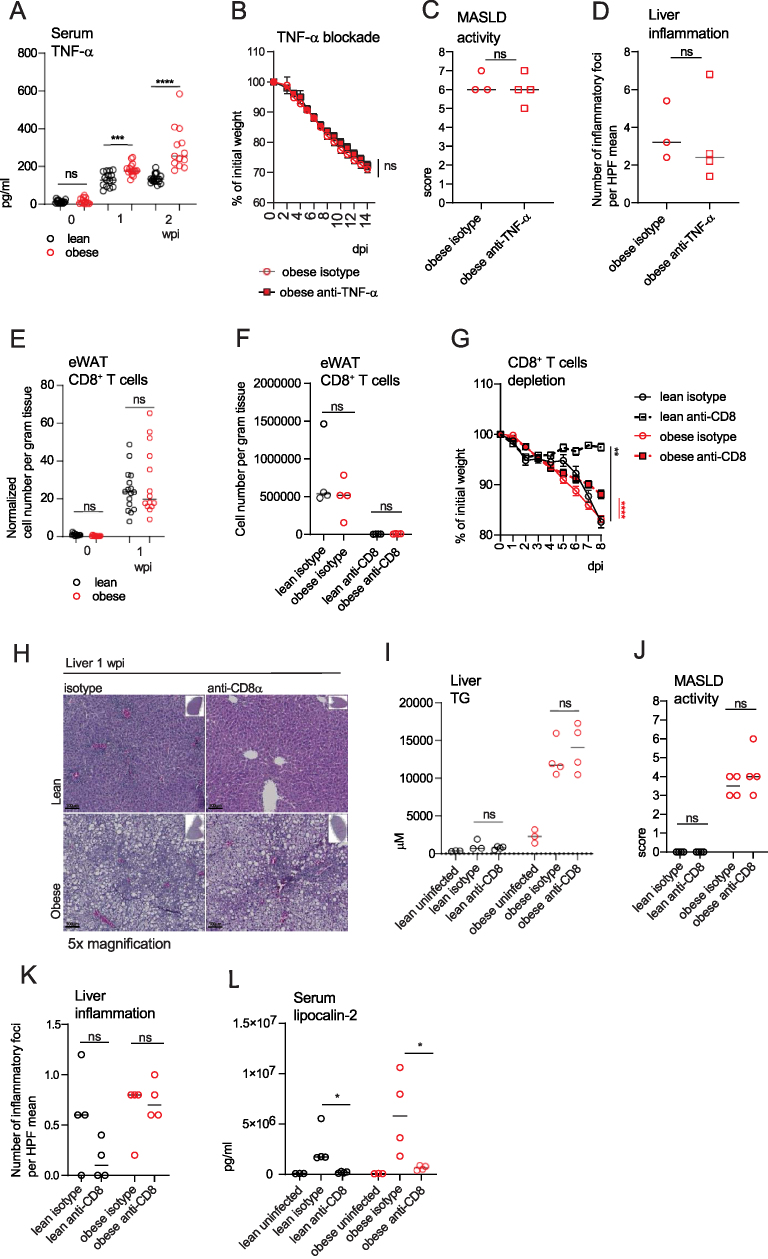
Effects of immunometabolic targeting strategies on infection-driven weight loss and on hepatic changes in lean and obese mice. Assessment of the involvement of TNF-α in infection-induced pathology (A–D); (A) TNF-α concertation in serum, (B) body weight trajectories, (C) MASLD activity score, and (D) hepatic immune infiltration quantified as number of inflammatory foci per high power field (HPF) post-infection in obese mice treated with isotype or anti-TNF-α antibodies. (E–L) Role of CD8^+^ T cells on pathology in LCMV-infected lean and obese mice. (E) Flow cytometric quantification of CD8^+^ T cell infiltration into the eWAT, (F) efficacy of anti-CD8α antibody in depleting CD8^+^ T cells assessed at week one post infection. (G) Body weight trajectories post-infection in infected CD8-T-cell-sufficient or -deficient lean and obese mice. (H) Representative histological evaluation of liver pathology assessed by H&E staining (5-times magnification) in the presence or absence of CD8^+^ T cell in infected lean or obese mice. Concentrations of (I) liver triglycerides (TG), (J) overall MASLD activity score, (K) hepatic immune infiltration per high power field (HPF) mean, (L) concentrations of the systemic inflammation marker lipocalin-2 in the presence or absence of CD8^+^ T cell in infected lean or obese mice. dpi – days post infection, wpi – weeks post infection. Each dot represents (A, C–F, I-L) one biological replicate or (B, G) average value of the pool of four to eight mice. Error bars represent mean ± SEM. Statistical significance was determined using two-tailed Student's t-tests for comparisons between two groups and mixed-effects model for repeated measures analyses. ∗p < 0.05, ∗∗p < 0.01, ∗∗∗p < 0.001, ∗∗∗∗p < 0.0001, ns, not significant. Related to Supplementary Fig. 3.

TNF-α regulates multiple aspects of adipose tissue homeostasis and has been implicated in pathological weight loss under inflammatory settings [24,[37], [38], [39]]. LCMV infection induced a surge in TNF-α in lean and obese mice, with the obese group showing significantly higher levels as compared to their lean counterparts (Figure 3A). To determine whether TNF-α contributes to the observed weight loss and liver pathology in obese mice, we neutralized this cytokine using a TNF-α–blocking antibody. To minimize potential interference of anti-TNF-α treatment with the priming and expansion of anti-viral T cells [40,41], we administered TNF-α blocking antibody from day seven post infection and monitored weight loss until two weeks post infection. However, weight loss, the mass of eWAT and iWAT, as well as MASLD activity and hepatic immune infiltration in obese mice remained unaffected by cytokine blockade (Figure 3B–D, Supplementary Fig. 3A and B), indicating that TNF-α is not a primary driver of infection-induced weight loss/fat depletion, aligning with previous findings in lean animals [7], and it is not the main driver of liver pathology in this context. However, given our window of intervention, we cannot exclude a potential role for TNF-α during the early phase of the infection.

### 2.5 Pre-existing hyperglycemia in obese mice does not drive infection-induced weight loss

Disruptions in glucose homeostasis may contribute to WAT dysfunction and promote fat depletion in inflammatory contexts [42,43]. To examine whether pre-existing hyperglycemia in obese animals may have contributed to the exaggerated weight loss, we normalized blood glucose concentrations in obese mice prior to the infection by administration of the well-established anti-diabetic drug metformin and continued with the medication until the end point of the experiment. However, despite the normalization of blood glucose levels prior to infection (Supplementary Fig. 3C and D), metformin-treated animals exhibited a degree of weight loss comparable to saline-treated controls (Supplementary Fig. 3E), suggesting that neither preexisting hyperglycemia nor metformin treatment influenced the weight loss trajectory.

### 2.6 CD8^+^ T cells are not the main driver of infection-induced weight loss in obese mice

CD8^+^ T cells are key mediators of LCMV-induced weight loss in lean mice [7,9]. Thus, we addressed the role of these cells in LCMV-induced WAT and liver pathology in obese mice. LCMV triggered a progressive accumulation of both total and virus-specific CD8^+^ T cells in WAT at one- and two-weeks post infection (Figure 3E, Supplementary Fig. 3F–I), suggesting a role for these cells in regulating WAT homeostasis during infection, and confirming that WAT represents a reservoir for antigen-specific T cells during viral infection [44]. Notably, while CD8^+^ T cell accumulation was comparable between lean and obese mice in the iWAT, it was reduced in the eWAT of obese mice at two weeks post-infection, mirroring the numbers of CD45^+^ cells across these depots (Supplementary Fig. 3F–I). To evaluate the contribution of CD8^+^ T cells to infection-induced weight loss, we depleted CD8^+^ T cells using an anti-CD8α depleting antibody prior to infection. This approach effectively eliminated CD8^+^ T cells from WAT (Figure 3F, Supplementary Fig. 3J). In line with previous findings, CD8^+^ T cell depletion fully prevented infection-induced weight loss in lean mice (Figure 3G) [7]. In contrast, obese mice were only partially protected (Figure 3G). This partial protection points towards CD8^+^ T cell–independent mechanisms, such as macrophages (Supplementary Fig. 4), responsible for the infection-induced weight loss, although their exact role in this pathology warrants further examination.

### 2.7 Infection-induced MASLD activity and liver inflammation in obese mice is independent of CD8^+^ T cells

Given the modest attenuation of infection-induced weight loss observed with CD8^+^ T cell depletion, and previously reported key role of CD8^+^ T cells in LCMV-induced liver pathology [21], we sought to further investigate the role of CD8^+^ T cells in liver pathology. The depletion of CD8^+^ T cells did not alter overall hepatic steatosis, TG content, or MASLD activity in either lean or obese animals (Figure 3H–J). This is in line with the sustained profound weight loss in obese animals upon CD8^+^ T cells depletions (Figure 3G). Nevertheless, the absence of CD8^+^ T cells reduced, albeit not significantly, the density of inflammatory foci in the livers of lean but not in obese mice (Figure 3K). In contrast, systemic levels of lipocalin-2 decreased in the absence of CD8^+^ T cells in both groups (Figure 3L). Collectively, these findings suggest that the hepatic pathology observed in obese mice is independent of CD8^+^ T cells, but absence of these cells mitigates systemic inflammation.

In summary, this study demonstrates distinct responses to chronic viral infection between lean and obese hosts and reveals that the mechanisms underlying infection-induced pathology depend on host metabolic status. These findings underscore the need to explore the mechanisms driving chronic virus–induced immunopathology in obese hosts to identify therapeutic targets that limit virus-induced tissue damage.

## 3 Materials and methods

### 3.1 Mice and LCMV infection

C57BL/6J male mice were purchased from the Jackson Laboratory. Mice were maintained under SPF conditions at 22 °C (±1 °C) at the animal facility, University of Copenhagen, and housed in groups of four. Alternatively, they were single housed when monitored for food and water intake, activity and temperature. The animals were fed Rodent Diet with 60% kcal% fat (Research Diets, D12492) or standard chow diet (Brogaarden, Altromin 1310) for 12–16 weeks prior to infection. Mice received 2 × 10^6^ PFU of LCMV clone-13 intravenously to initiate chronic infection. Viral titers were determined in kidney lysates by plaque assay as previously described [45]. All experiments were conducted in accordance with the national regulations under ethical license 2020-15-0201-00586.

### 3.2 *In vivo* treatments

Metformin (PHR1064, Merck) was administered at 250 mg/kg body weight orally prior to the infection, followed by daily intraperitoneal injections at 50 mg/kg body weight until the endpoint of the experiment. For antibody *in vivo* treatments, the following antibodies from BioXcell were used: anti-TNF-α (XT3.11, BE0058), anti-CD8a (YTS169.4, BE0117), IgG1 isotype (BE0083) and IgG2b isotype (BE0090). Anti-TNF-α neutralizing antibodies or IgG1 isotype control were intraperitoneally injected (à 0.5 mg) on day seven post infection and re-administered every second day up to 15 days post-infection. For CD8 T cell-depletion, 0.4 mg of anti-CD8a was injected intraperitoneally one day prior to LCMV infection, with additional doses of 0.2 mg on days one and four post infection. FACS analysis was performed on adipose tissue to confirm CD8^+^ T cell depletion.

### 3.3 Measurements of ketones and glucose

Circulating glucose and ketone bodies (β-hydroxybutyrate) were measured with FreeStyle Precision Neo and ContourXT (Bayer) strips, respectively, by sampling tail blood at noon, 5h after food removal.

### 3.4 Enzymatic assays

Lipocalin-2, alanine aminotransferase (ALT) was measured in serum by ELISA (R&D Systems and Abcam, respectively), and the hepatic concentrations of triglycerides in liver tissue was evaluated using the Triglyceride-Glo™ Assay (Promega) following manufacturer instructions.

### 3.5 Measurements of core body temperature and gross motor activity

The measurement of body temperature and gross motor activity were performed by the Rodent Metabolic Phenotyping Platform at the Novo Nordisk Foundation Center for Basic Metabolic Research. Body temperature and motor activity were measured with radio frequency identification temperature transponders (UCT-2112 microchips, Unified Information Devices), which were implanted into the peritoneal cavity. In brief, mice were anaesthetized with isoflurane and received 5 mg kg−1 Rimadyl (ScanVet, 027693) and 8 mg kg−1 Lidocaine (AstraZeneca). The sterile probe was inserted into the peritoneum via a midline incision. After closure, mice were allowed to recover on a heated surface and received 5 mg kg−1 Rimadyl for 3 consecutive days. Mice were allowed to recover for at least 7 days before study started. E-mitter data was recorded by placing ER4000 Receivers under the cages. Temperature data was integrated in the Phenomaster software. RFID temperature transponder data was recorded by using UID Mouse Matrix system (Unified Information Devices).

### 3.6 Mouse tissue extract preparation and immunoblotting

Harvested organs were snap frozen in liquid nitrogen and powdered using Covaris CP02 tissue crasher. Lysates were prepared as previously described [46], however exchanging the lysis buffer to detergent-free lysis buffer for initial lysate preparations (Tris–HCl (50 mM), EDTA (1 mM), EGTA (1 mM), Sucrose (0.27 M), Glycerol-2-phosphate disodium (20 mM), NaF (50 mM), Na_4_P_2_O_7_·10H_2_O (5 mM), PMSF (0.5 mM), benzamidine HCl (1 mM), leupeptin (1 μg/ml), pepstatin A (1 μg/ml), DTT (1 mM), microcystin-LR (1 mM), Na_3_VO_4_ (1 mM)). Bradford assay was used to quantify protein concentration in clarified lysates using Bradford reagent (ThermoFisher) and bovine serum albumin (BSA) as standard. Lysates were denatured in Laemmli buffer at 95 °C for 5 min in the presence of 100 mM DTT, and the proteins were separated by tris-glycine SDS-PAGE (4–20% gradient gels: Criterion™ TGX Stain-Free™ or homemade, BioRad) followed by transfer onto nitrocellulose membrane in tris-glycine buffer (48.2 mM Tris, 40 mM glycine). Membranes were blocked for at room temperature 45 min in 3% skimmed milk supplemented in TBS-T (20 mM tris–HCl pH 7.5, 137 mM NaCl, 0.1% (v/v) Tween-20) and subsequently incubated with primary antibodies diluted in TBS-T/4% BSA/0.0025% sodium azide overnight. The following primary antibodies (Cell Signaling Technology) were used: ACC (3676), Vinculin (13901T), HSL [D6W5S] XP® (18381T), ATGL [30A4] (2439S), CPT2 (52552), UCP-1 (Abcam, ab10983). Next, membranes were washed in TBS-T three times followed by incubation with Affinipure Donkey Anti-Rabbit IgG (H + L) HRP-conjugated secondary antibodies (Jackson immuno research, 711-035-152) diluted 1:10000 for 45 min, followed by three washes prior to detection. Chemiluminescent HRP substrate (Immobilion Western, WBKLS5000) was applied onto the membranes, which were subsequently imaged on the Odyssey® XF imaging system (Li-COR) and analyzed by Image Studio Lite v 5.2. Total protein was quantified by Revert 700 Total Protein Stain.

### 3.7 Histology and assessment of liver histopathology

Liver (left lobe) and WAT were fixed in 10% formaline for 24h. Tissues were perfused and embedded in paraffin and cut into 3.5 μm sections. Tissue sections were stained with hematoxylin and eosin (H&E) for histological assessment. Images were collected using Zeiss Axioscan Z1 scanner and analysis was performed using QuPath v0.5.1 software or manually by assessment of the entire liver slide for MASLD activity score and a minimum of five randomly chosen view fields (1mm2 each) per specimen for assessment of inflammatory burden. An average of the quantified foci across chosen view fields was reported. MASLD activity scoring was assessed blindly on H&E-stained liver sections using a previously described scoring system [47] adapted from [48]. Briefly, the hepatocellular hypertrophy, macrovesicular, and microvesicular steatosis were scored relative to the total liver area (<5% = 0; 5%–33% = 1; 33%–66% = 2; and >66% = 3). Inflammation was scored by quantifying the number of inflammatory foci per 1 mm^2^ and presented as average of five areas per liver.

### 3.8 Cell isolation

WAT immune cell isolation was performed by enzymatic digestion. WAT was cut into small pieces and placed in digestion beakers containing 2.5 ml media (DMEM/10 mM HEPES/1 mM sodium pyruvate/1x MEM-NEAA/10% FCS/PenStrep) supplemented with Liberase TM (0.3 Wuensch/ml, Merck) and DNAse I (1 mg/ml, Merck). The samples were placed in water bath at 37 °C and incubated for 40′ under continuous shaking. Samples were vortexed and supplemented with 10 ml ice-cold media, centrifuged for 5′ at 500g. The pellets were resuspended in ice-cold media and filtered through 70um cell strainers into tubes containing ice-cold media, washed and centrifuged for 5’ at 500g. The cell pellet was prepared for flow cytometric staining.

### 3.9 Flow cytometry

Samples were blocked in 10% rat serum and 1:300 CD16/CD32 FcR block for 15min, followed by washing and staining with antibody mix for 20’ at 4 °C. Cells were washed in PBS and fixed using 2% formaldehyde/PBS solution or the FoxP3 Fixation/Permeabilization Kit (eBioscience) following manufacturer instructions. Dead cells identified as fixable Viability Dye eFluor®780 (eBioScience), and cell aggregates (identified on FSC-A versus FSC-W scatterplots) were excluded from analyses. Cells were quantified with Accucount fluorescent particles (7.4um, Spherotech). Serum TNF-α was measured with the LEGENDplexTM Mouse Anti-Virus Response Panel (13-plex) (Biolegend, 740622). Antibodies (BioLegend) and tetramers used in this study: anti-CD8β PE (clone YTS156.7.7), anti-CD45.2 A700 (clone 30-F11), anti-CD19 FITC (clone 6D5), anti-CD90.2/Thy1.2 FITC (clone 30-H12), anti-CD11b BV605 (clone M1/70), anti-CD11c PE-Cy7 (clone N418), anti-CD64 BV421 (clone X54-5/7.1), anti-Ly6C PerCP/Cy5.5 (clone HK1.4), anti-Ly6G PE (clone 1A8), anti-MHCII BV650 (clone M5/114.15.2), anti-Tim-4 Alexa Fluor 647 (clone RMT4-54), anti-F4/80 BV510 (clone BM8), anti-CD206 PE-Dazzle 594 (clone C068C2), anti-Siglec F PE (BD Biosciences, clone E50-2440), APC-coupled H-2Db/LCMV.GP33 (KAVYNFATM) tetramer (MHC Tetramer Production Facility - Baylor College of Medicine (Tx, USA)). All samples were acquired on a Beckman Coulter CytoFLEX flow cytometer and analyzed using FlowJo software v10.10.0 (Tree Star) or the LEGENDplex™v8 Data Analysis Software.

### 3.10 Statistical analysis

Data are expressed as mean ± SEM unless otherwise indicated. Statistical analyses were performed using two-tailed unpaired student's t-test, mixed-effects models for repeated measures, and two-way ANOVA with Tukey post hoc correction test as appropriate, using GraphPad Prism v10.4.1 (La Jolla, CA, USA). p values of <0.05 were considered statistically significant.

## CRediT authorship contribution statement

**Katarzyna M. Luda:** Writing – review & editing, Writing – original draft, Project administration, Methodology, Investigation, Funding acquisition, Formal analysis, Data curation, Conceptualization. **Marianne Agerholm:** Writing – review & editing, Project administration, Investigation. **Si Brask Sonne:** Investigation. **Dipsikha Biswas:** Writing – review & editing, Investigation. **Lisa DeCamp:** Project administration, Investigation. **Russell G. Jones:** Writing – review & editing, Supervision. **Allan Randrup Thomsen:** Writing – review & editing, Supervision. **Benjamin Anderschou Holbeck Jensen:** Writing – review & editing, Supervision, Investigation. **Jan Pravsgaard Christensen:** Writing – review & editing, Supervision, Investigation. **Kei Sakamoto:** Writing – review & editing, Supervision, Funding acquisition.

## Funding

This project was supported by the Marie Skłodowska-Curie Actions Postdoctoral Fellowship, Grant Agreement MSCA-HE-2021 No. 101063888, Acronym: Virobe. The study was also supported by the funding from the 10.13039/501100009708Novo Nordisk Foundation (Grant numbers: NNF18CC0034900, NNF23SA0084103).

## Declaration of competing interest

The authors declare that they have no known competing financial interests or personal relationships that could have appeared to influence the work reported in this paper.

## Data Availability

Data will be made available on request.

## Appendix A Supplementary data

Figure S1 Changes in WAT induced by chronic viral infection in lean and obese mice. Comparison of indicated parameters between infected or uninfected lean and obese mice in the course of infection. (A) Total body weight before infection, daily monitoring of (B) water consumption, (C) mouse activity in the cage, (D) body temperature. (E) Representative pictures of iWAT. (F) Flow cytometric quantification of immune cells (CD45^+^) in eWAT and iWAT. (G-H) WB analysis of (G) iWAT and eWAT lysates assessing total protein per WB lane, and (H) expression of proteins involved in lipid metabolism in iWAT. dpi – days post infection, wpi – weeks post infection. (I-J) Immunoblots and quantification of (I) total protein per WB lane, and (J) UCP-1 and CPT2 expression in interscapular brown adipose tissue (BAT). Each data point represents (B-D) average value of the pool of at least five mice or (A, F-J) each dot and WB band represents one biological replicate. Error bars represent mean ± SEM. Statistical significance was determined using two-tailed Student's t-tests for comparisons between two groups and mixed-effects model for repeated measures analyses. ∗p < 0.05, ∗∗p < 0.01, ∗∗∗p < 0.001, ∗∗∗∗p < 0.0001, ns, not significant. Related to Figure 1.

Figure S2 Western Blot quality control in liver protein loading. (A) Total protein staining in WB from liver samples. Each lane represents one biological replicate. Related to Figure 2.

Figure S3 Effects of immunometabolic targeting strategies on infection-driven weight loss and on hepatic changes in lean and obese mice. Comparison of indicated parameters between lean and obese mice in the course of infection. Comparison of weights (A) eWAT and (B) iWAT two weeks post infection in isotype or anti-TNF-α treated obese mice. (C-E) Evaluating involvement of pre-existing hyperglycemia in infection-induced weight loss. (C-D) Impact of metformin treatment on fasted blood glucose concentration in uninfected lean and obese mice (C) prior to and (D) at the day of infection; (E) body weight trajectories post-infection in infected obese mice treated with saline or metformin. (F-J) Flow cytometric quantification of (F, H) CD8^+^ and (G, I) GP33-tetramer stained CD8^+^ T cell harvested from (F-G) eWAT and (H–I) iWAT. Quantification was expressed as fold change of cell numbers per gram of tissue relative to the mean value of uninfected lean mice. (J) Representative flow cytometry plot confirming CD8^+^ T-cell depletion in eWAT. (A-D, F–I) Each dot represents one biological replicate, or (E) average value of the pool of at least five mice. Error bars represent mean ± SEM. Statistical significance was determined using two-tailed Student's t-tests for comparisons between two groups and mixed-effects model for repeated measures analyses. ∗p < 0.05, ∗∗p < 0.01, ∗∗∗p < 0.001, ∗∗∗∗p < 0.0001, ns, not significant. Related to Figure 3.

Figure S4 Analysis of macrophage population in eWAT. (A-C) Flow cytometric analysis representing (A) gating strategy for identification of eWAT macrophages from cells isolated from uninfected lean mice, defined as single, live CD45.2^+^ Lin^−^ (CD19, CD90.2, Siglec-F, Ly6G) CD11b^+^ F4/80^+^ MHCII^+^ CD64^+^ cells, (B) representative flow cytometry plots showing expression of Tim-4 and CD206 on eWAT macrophages, and (C) Quantification of eWAT macrophages and the frequency of Tim-4^+^ cells within the macrophage population. (A-C) Each dot represents one biological replicate. Error bars represent mean ± SEM. Statistical significance was determined using two-tailed Student's t-tests for comparisons between two groups. ∗p < 0.05, ∗∗p < 0.01, ∗∗∗p < 0.001, ∗∗∗∗p < 0.0001, ns, not significant.
