# Supplementary material for: Chronic viral infection aggravates white adipose tissue dysfunction and liver pathology in obesity
Source: Mol Metab. 2026 Jun 9;110:102394. doi: 10.1016/j.molmet.2026.102394 (PMC13316306; doi:10.1016/j.molmet.2026.102394)
Supplement: Figure S1 — Changes in WAT induced by chronic viral infection in lean and obese mice. Comparison of indicated parameters between infected or uninfected lean and obese mice in the course of infection. (A) Total body weight before infection, daily monitoring of (B) water consumption, (C) mouse activity in the cage, (D) body temperature. (E) Representative pictures of iWAT. (F) Flow cytometric quantification of immune cells (CD45+) in eWAT and iWAT. (G-H) WB analysis of (G) iWAT and eWAT lysates assessing total protein per WB lane, and (H) expression of proteins involved in lipid metabolism in iWAT. dpi – days post infection, wpi – weeks post infection. (I-J) Immunoblots and quantification of (I) total protein per WB lane, and (J) UCP-1 and CPT2 expression in interscapular brown adipose tissue (BAT). Each data point represents (B-D) average value of the pool of at least five mice or (A, F-J) each dot and WB band represents one biological replicate. Error bars represent mean ± SEM. Statistical significance was determined using two-tailed Student's t-tests for comparisons between two groups and mixed-effects model for repeated measures analyses. ∗p < 0.05, ∗∗p < 0.01, ∗∗∗p < 0.001, ∗∗∗∗p < 0.0001, ns, not significant. Related to Figure 1. [file mmc1.docx]

# Supplementary figure 1

A Body weigth

✱✱✱✱


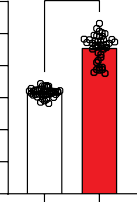
60

50

40

30

20

10

gram

0

lean obese

uninfected


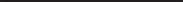
E iWAT

0 1 2 wpi

# B

12


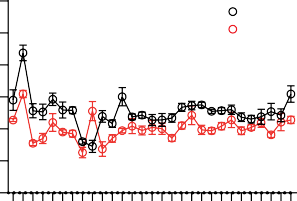


lean obese

10

8

6

4

2

gram

0

Water intake

# F

0

1

2

3

4

5

6

7

8

9

10

11

12

13

14

15

16

17

18

19

20

21

22

23

24

25

26

27

28

eWAT

dpi

# C

1000 Activity


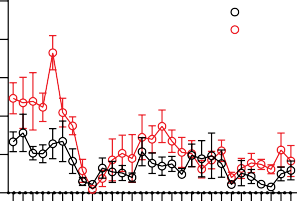

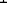


lean obese

800

600

ns

400

activity index

200

0

0

1

2

3

4

5

6

7

8

9

10

11

12

13

14

15

16

17

18

19

20

21

22

23

24

25

26

27

28

iWAT

dpi

# D

40.0

37.5

35.0

Degree Celsius

32.5

ns

30.0

iWAT

Temperature

ns

dpi


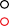

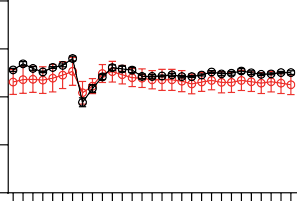


lean obese

0

1

2

3

4

5

6

7

8

9

10

11

12

13

14

15

16

17

18

19

20

21

22

23

24

25

26

27

28


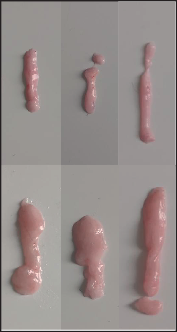
1×107

8×106

6×106

Total cell number

4×106

Lean

2×106

0


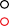


CD45+ cells

0 1 2


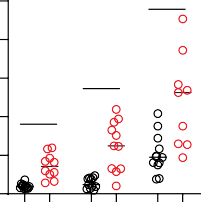


**

****

****

wpi

lean obese

4×106

3×106

Total cell number

2×106

1×106

0


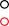


CD45+ cells

0 1 2


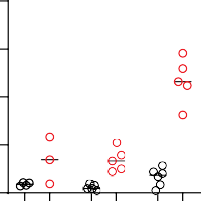


****

ns

***

wpi

lean obese

1×107

8×106

Cell number per gram tissue

6×106

4×106

2×106

0


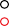


CD45+ cells

0 1 2


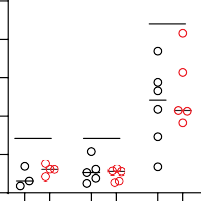


ns

ns

ns

wpi

lean obese

# G

Obese

eWAT total protein staining

H iWAT

uninfected 1 week post infection


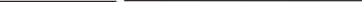


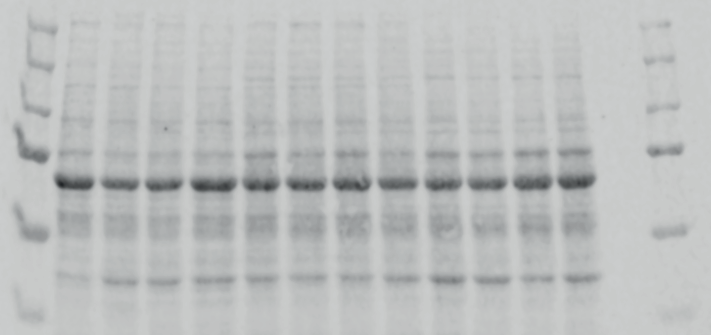
L 1 2 3 4 5 6 7 8 9 10 11 12 E L

Loading pattern for eWAT and iWAT:

1, 2: lean uninfected

3, 4: obese uninfected

5-8: lean 1wpi

9-12: obese 1wpi L: ladder lane

E: empty lane

Vinculin ACC HSL

## ATGL CPT2

lean obese lean obese


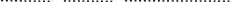

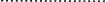


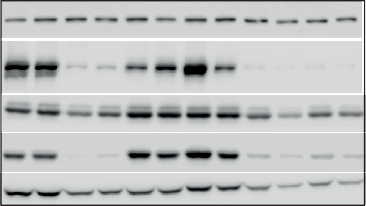
iWAT total protein staining

L 1 2 3 4 5 6 7 8 9 10 11 12 L L


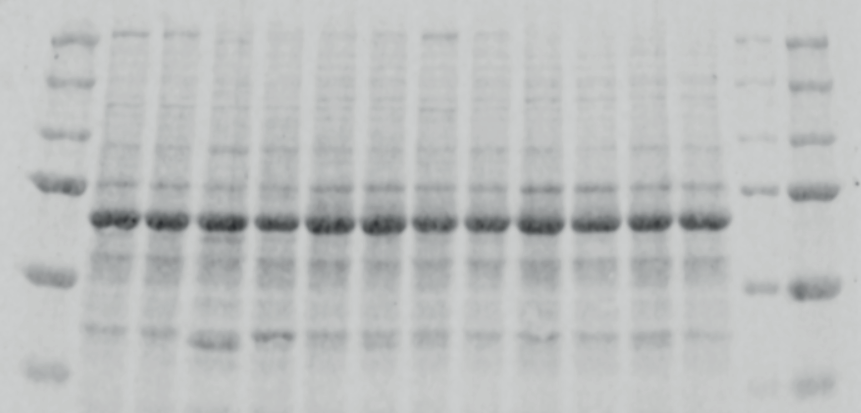


250

200

150

100

50

Signal

0

iWAT


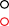
total protein

iWAT ACC


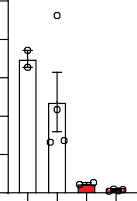
10

8

6

4

2

Signal

0

2.0

1.5

1.0

0.5

Signal

0.0

iWAT HSL


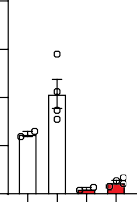


iWAT ATGL


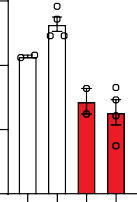
15

10

5

Signal

0

iWAT


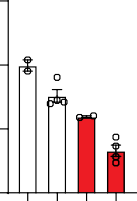
15 CPT2

10

5

Signal

0

lean obese


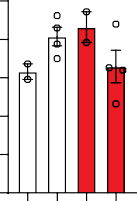
0 1 0 1 0 1

0 1 0 1

0 1 0 1

0 1 0 1 0 1

wpi

# I J

wpi

wpi

wpi

wpi

## BAT BAT

lean

obese

lean

obese

uninfected 2wpi uninfected 2wpi

Vinculin CPT2 UCP-1


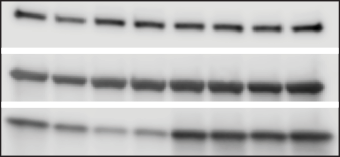
uninfected 2wpi uninfected 2wpi


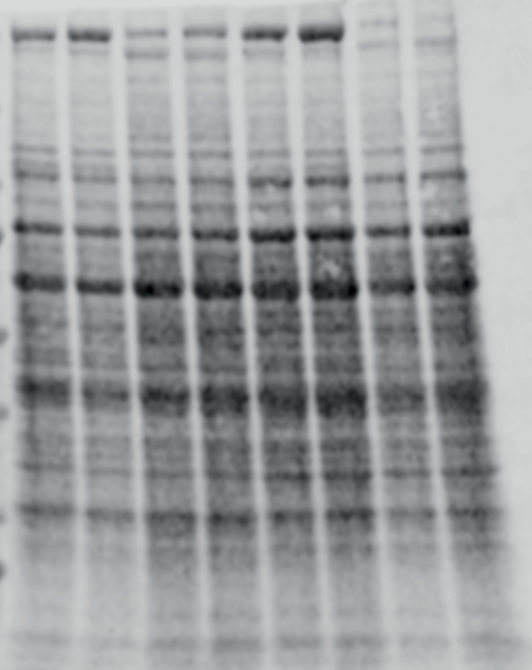
800

600

400

200

## BAT

80

60

40

20

Signal

## BAT

80

60

40

20

Signal

## BAT

0


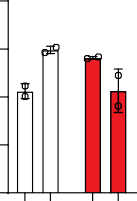


total protein

Signal

wpi: 0 2 0 2

0

wpi: 0 2 0 2


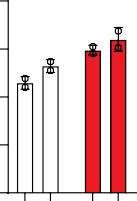


CPT2

0

wpi: 0 2 0 2


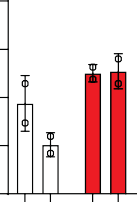


UCP-1

lean obese

lean obese lean obese
