# Supplementary material for: Chronic viral infection aggravates white adipose tissue dysfunction and liver pathology in obesity
Source: Mol Metab. 2026 Jun 9;110:102394. doi: 10.1016/j.molmet.2026.102394 (PMC13316306; doi:10.1016/j.molmet.2026.102394)
Supplement: Figure S2 — Western Blot quality control in liver protein loading. (A) Total protein staining in WB from liver samples. Each lane represents one biological replicate. Related to Figure 2. [file mmc2.docx]

Supplementary figure 2

A

# Liver total protein staining

L 1 2 3 4 5 6 7 8 9 10 11 12

13 14 15 16 17 18 19 20 21 22


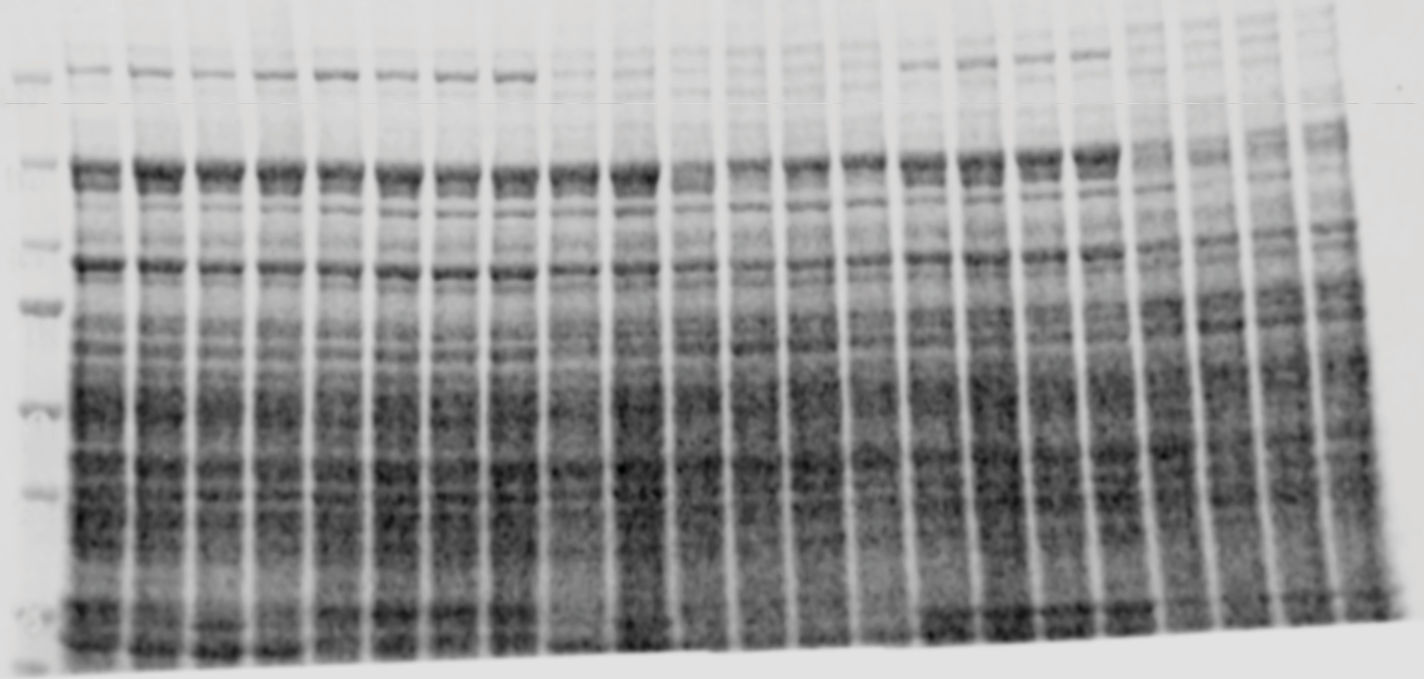
Loading pattern: L: ladder lane

1-4: lean uninfected

5-8: obese uninfected

9-10: lean 1 wpi

11-14: obese 1 wpi

15-18: lean 1wpi

19-22: obese 2wpi
