# Supplementary material for: Chronic viral infection aggravates white adipose tissue dysfunction and liver pathology in obesity
Source: Mol Metab. 2026 Jun 9;110:102394. doi: 10.1016/j.molmet.2026.102394 (PMC13316306; doi:10.1016/j.molmet.2026.102394)
Supplement: Figure S3 — Effects of immunometabolic targeting strategies on infection-driven weight loss and on hepatic changes in lean and obese mice. Comparison of indicated parameters between lean and obese mice in the course of infection. Comparison of weights (A) eWAT and (B) iWAT two weeks post infection in isotype or anti-TNF-α treated obese mice. (C-E) Evaluating involvement of pre-existing hyperglycemia in infection-induced weight loss. (C-D) Impact of metformin treatment on fasted blood glucose concentration in uninfected lean and obese mice (C) prior to and (D) at the day of infection; (E) body weight trajectories post-infection in infected obese mice treated with saline or metformin. (F-J) Flow cytometric quantification of (F, H) CD8+ and (G, I) GP33-tetramer stained CD8+ T cell harvested from (F-G) eWAT and (H–I) iWAT. Quantification was expressed as fold change of cell numbers per gram of tissue relative to the mean value of uninfected lean mice. (J) Representative flow cytometry plot confirming CD8+ T-cell depletion in eWAT. (A-D, F–I) Each dot represents one biological replicate, or (E) average value of the pool of at least five mice. Error bars represent mean ± SEM. Statistical significance was determined using two-tailed Student's t-tests for comparisons between two groups and mixed-effects model for repeated measures analyses. ∗p < 0.05, ∗∗p < 0.01, ∗∗∗p < 0.001, ∗∗∗∗p < 0.0001, ns, not significant. Related to Figure 3. [file mmc3.docx]

# Supplementary figure 3

A

1500

1000

500

eWAT

# B

1000

800

600

400

200

iWAT

0 0


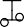


2 wpi

ns


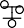


2 wpi

ns

mg

mg


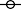
 obese isotype


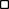
 obese anti-TNF-α


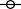
 obese isotype


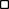
 obese anti-TNF-α

# C

Blood

20 fasted glucose


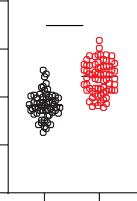


****

15

10

5

0

mM

lean uninfected obese uninfected

# D

15.0

12.5

10.0

7.5

5.0

2.5

mM

0.0

Blood

fasted glucose

**

# E

110

100

% of initial weight

90

80

0

1

2

3

4

5

6

7

8

Metformin treatment

ns

dpi

obese saline obese metformin

# F

Cell number per gram tissue fold change above lean conrtrol

800

eWAT

CD8+ T cells

# G

4000

Cell number per gram tissue fold change above lean conrtrol

eWAT

CD8+GP33-tet+ cells

600 3000


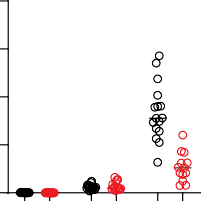


****

ns

ns


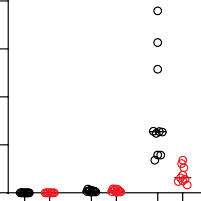


**

ns ns

400

200

0

# H

80

Cell number per gram tissue fold change above lean conrtrol

0 1 2

wpi

lean

obese

iWAT

CD8+ T cells

2000

1000

0

# I

Cell number per gram tissue fold change above lean conrtrol

1500

0 1 2

wpi

lean obese

iWAT

CD8+GP33-tet+ cells

ns

60


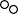

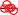

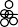

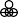


ns

ns

ns

ns

ns

1000

40

500

20

0

0 1 2

wpi

lean obese

0

0 1 2

wpi

lean obese

J eWAT - gated on live single cells

Isotype control anti-CD8α


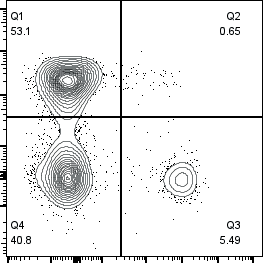

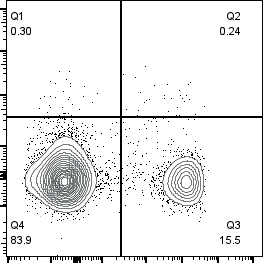


CD4

CD8β
