# Supplementary material for: Chronic viral infection aggravates white adipose tissue dysfunction and liver pathology in obesity
Source: Mol Metab. 2026 Jun 9;110:102394. doi: 10.1016/j.molmet.2026.102394 (PMC13316306; doi:10.1016/j.molmet.2026.102394)
Supplement: Figure S4 — Analysis of macrophage population in eWAT. (A-C) Flow cytometric analysis representing (A) gating strategy for identification of eWAT macrophages from cells isolated from uninfected lean mice, defined as single, live CD45.2+ Lin− (CD19, CD90.2, Siglec-F, Ly6G) CD11b+ F4/80+ MHCII+ CD64+ cells, (B) representative flow cytometry plots showing expression of Tim-4 and CD206 on eWAT macrophages, and (C) Quantification of eWAT macrophages and the frequency of Tim-4+ cells within the macrophage population. (A-C) Each dot represents one biological replicate. Error bars represent mean ± SEM. Statistical significance was determined using two-tailed Student's t-tests for comparisons between two groups. ∗p < 0.05, ∗∗p < 0.01, ∗∗∗p < 0.001, ∗∗∗∗p < 0.0001, ns, not significant. [file mmc4.docx]

# Supplementary figure 4

A

eWAT (uninfected lean mice)

Single live cells Leukocytes


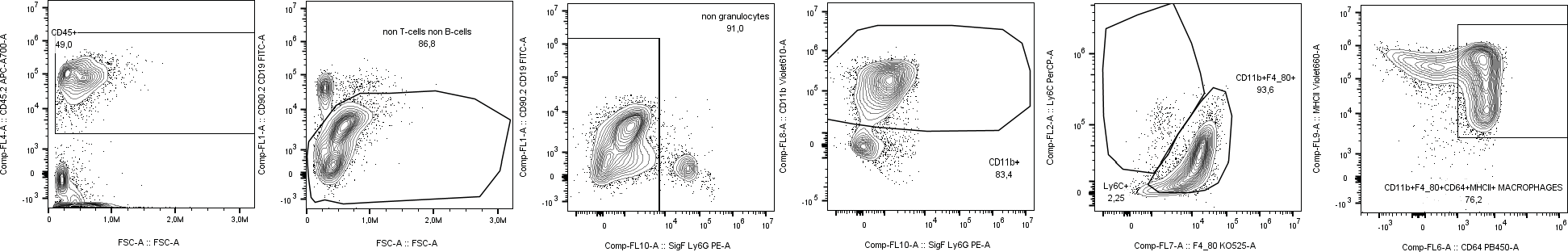


FSC-A

FSC-A

SiglecF, Ly6G

SiglecF, Ly6G

F4/80

CD64

CD11b+F4/80+

CD64+MHCII+ (macrophages)

CD11b+

CD11b+ F4/80+

non-lymphoid cells

Leukocytes

non-granulocytes

CD45.2

CD19, CD90.2

CD19, CD90.2

CD11b

Ly6C

MHCII

non-lymphoid cells non-granulocytes CD11b+ F4/80+CD11b+

# B C

Gated on eWAT macrophages (CD11b+F4/80+CD64+MHCII+)

lean obese

uninfected


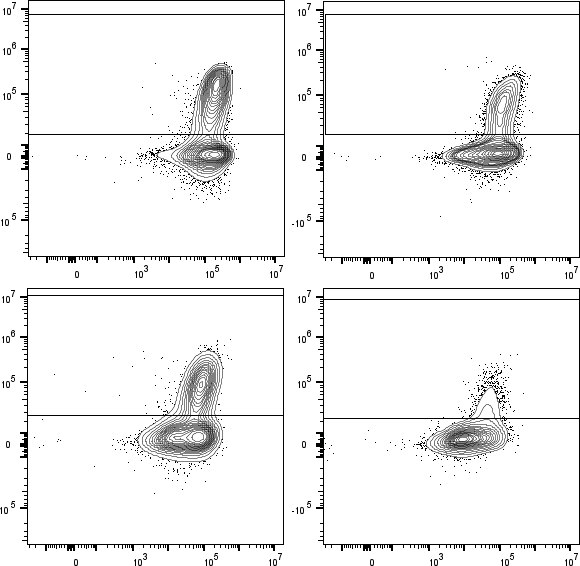


Tim-4+ 44.5%

Tim-4+ 24.8%

Tim-4+ 36.6%

Tim-4+ 8.22%

1×107

8×106

Number of CD11b+F4/80+CD64+

MHCII+ per g tissue

6×106

4×106

eWAT macrophages


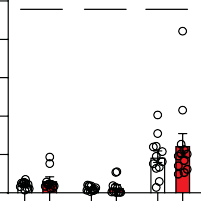


ns

ns

ns

2×106

0

0 1 2

wpi

lean obese

1 wpi

eWAT

% Tim-4+ macrophages

60


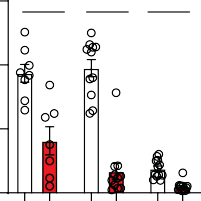


** ****

****

40

% Tim-4+ among CD11b+F4/80+CD64+

MHCII+ macrophages

20

Tim-4

## CD206

0

0 1 2

wpi

lean obese
